# MiRNA-202-5p promotes Colorectal Carcinogenesis through suppression of PTEN

**DOI:** 10.7150/jca.56186

**Published:** 2021-03-31

**Authors:** Jing Huang, Yaqin Zhang, Yuan Xu, Qi Xie, Shuang Wu, Yi Dong, Bing Chen, Yang Xia, Lili Guo, Qun Li, Hao Gu, Wanglai Hu

**Affiliations:** 1 Department of Immunology, the school of Basic Medical Sciences, Anhui Medical University, Hefei, China, 230032.; 2 Translational Research Institute, Henan Provincial People's Hospital, Molecular Pathology Center, Academy of Medical Science, Zhengzhou University, Zhengzhou, China, 450003.

**Keywords:** miR-202-5p, PTEN, c-Myc, CRC

## Abstract

Colorectal cancer (CRC) is still one of the leading causes of cancer-associated death in the modern society. The biological function of miR-202-5p for CRC development remains controversial, largely due to the fact that miR-202-5p can be tumor-suppressive or oncogenic in different contexts. Obtained results indicated that aberrant expression of miR-202-5p was observed in majority of human CRC samples and miR-202-5p was transcriptionally up-regulated by c-Myc. In addition, miR-202-5p functions to promote the activation of PI3K/Akt signaling pathway by directly suppressing PTEN. Silencing or enforced expression of miR-202-5p resulted in CRC cell proliferation inhibition and enhancement, respectively. Importantly, decreased PTEN level and increased phosphorylation of Akt were frequently associated with elevated miR-202-5p expression in colorectal cancer tissues. Increased miR-202-5p expression may serve as a tumor promoter by directly targeting PTEN in colorectal cancer.

## Introduction

Colorectal cancer is the world's third most deadly cancer killing almost 700,000 people annually 1, 2. The pathogenesis of colorectal cancer (CRC) is still not clearly understood despite important progress being made in diagnostic and therapeutic strategies 3, 4. Therefore, the clarification of the molecular mechanisms that underlying colorectal carcinogenesis is instantly needed 5, 6.

Accumulating evidences have suggested that aberrant expression of miRNAs plays a key role in the pathologic process of colorectal carcinogenesis 7. Several studies have reported that distinct miRNAs are associated with the progression of CRC by regulating of cell proliferation, apoptosis, differentiation, and aging 8-11. MiR-202-5p was first identified in human testis where it plays a key role in spermatogenesis 12-14. Subsequently, miR-202-5p was found to play critical roles in tumors. However, previous studies have conflicted on the role of miR-202-5p in tumorigenesis. Some studies have reported that it functions as a tumor suppressor in osteosarcoma, bladder cancer, and chronic myeloid leukemia 8, 15, 16. On the other hand, miR-202-5p has also been shown to be up-regulated in multiple tumor types, such as neuroblastoma, testicular tumors, and breast cancers, thereby promoting tumorigenesis and drug resistance 12, 17, 18. The role of miR-202-5p in CRC, whether it is an oncogene or a tumor-suppressive gene, has not been elucidated.

Activation of phosphatidylinositol 3-kinase (PI3K) pathway is particularly important for CRC where its activation is negatively regulated by phosphatase and tensin homolog (PTEN) 19-21, which regulates cell proliferation, migration, and metabolism 22. Therefore, PTEN expression is tightly regulated in CRC. It's well recognized that both transcriptional and post-transcriptional mechanisms are involved in regulating PTEN expression 21, 23, 24. In addition, the miRNA-mediated down-regulation of PTEN represents an important post-translational mechanism for maintaining an optimal level PTEN during tumorigenesis 25, 26. For example, studies have reported that miR-21 directly targets PTEN in multiple types of human tumors, including hepatocellular carcinoma, lung cancers, and ovarian cancers 27, 28. Another compelling example is miR-25, which negatively regulates PTEN in melanoma 29. However, little is known about post-transcriptional miRNA-mediated regulation of PTEN in colorectal carcinogenesis.

In this study, we demonstrate that miR-202-5p is aberrantly upregulated in colorectal cancers, and its levels correlate with c-Myc expression and PI3K/Akt pathway activation. Mechanistically, miR-202-5p is transcriptionally up-regulated by c-Myc, and it functions as an oncogenic miRNA by targeting PTEN expression directly, thereby activating PI3K/Akt pathway and promoting cell cycle progression. Results obtained in this study established that miR-202-5p is essential for CRC cell proliferation, with practical implications of interference with miR-202-5p in colorectal cancer treatment.

## Materials and Methods

### Cell lines and patient tissue samples

The HCT116 and HEK293T cell lines were cultured in DMEM medium containing 10% FBS. Specimens from 25 patients who were diagnosed with colorectal cancer were collected from Henan Provincial People's Hospital of Zhengzhou University between 20^th^ Mar 2019 and 11^th^ Apr 2020. Samples were obtained by surgical excision and stored at -80 °C. All the experiments using human tissues were compliant with relevant ethical regulations and approvals were obtained from the Human Research Ethics Committees of Zhengzhou University.

### Luciferase reporter assay

Luciferase reporter assays were performed as previously described 30. MiR-202-5p promoter and 3'UTR of PTEN were cloned and inserted into pGL3 and pSICHECK2 vectors, respectively. HCT116 cells were transfected with indicated plasmids for 24 hours followed by measuring of the Firefly and Renilla luciferase activities using the Dual-luciferase reporter assay system. The relative luciferase activities were produced by normalizing the Firefly luciferase activity to Renilla luciferase activity.

### Chromatin immunoprecipitation assays

Chromatin immunoprecipitation (ChIP) assays were performed by using the ChIP kit according to the manufacturer's instructions with anti-c-Myc and anti-IgG, respectively. The bound DNA fragments were subjected to qPCR and semi-quantitative RT-PCR analyses using specific primers (Supplementary Table S1). Semi-quantitative RT-PCR products were then separated by gel electrophoresis on 2% agarose gel.

### Immunohistochemistry and image analysis

Immunohistochemistry was carried out as previously described 31, 32. Colorectal cancer tissues were fixed and paraffin-embedded. The tissues were cut into 5μm sections and subjected to antigen retrieval using microwave heating at 95 °C in citrate buffer. The sections were then incubated with the indicated antibodies (Supplementary Table S1) and visualized using DAB immunolabelling system (DAB-2031, MaxinBio, Fuzhou, China). The sections were counterstained with Haematoxylin (G1080, Solarbio, Beijing, China) followed by whole-slide scanning which was performed using the panoramic slide scanner II (3D HISTECH, Budapest, Hungary). PTEN et al immunohistochemistry was quantified in tissues using the intensity score. The intensity score was then quantified using Case Viewer soft version 2.3 (3D HISTECH, Budapest, Hungary).

### EdU proliferation assays

EdU cell proliferation assays were performed using an EdU assay kit according to the manufacturer's instructions. Briefly, HCT116 cells were transfected with miR-202-5p mimics or inhibitors. 48 hours after transfection, the cells were incubated with 50 mM EdU for another 2 hours. Apollo 567 fluorescent dye and Hoechst 33342 staining were carried out before the proportion of EdU positive cells was analyzed using an Olympus IX73 microscope.

### Colony formation assay

HCT116 cells bearing the desired treatment were seeded into 6-well plates at a density of 2000 cells per well. The cells were allowed to grow for 14 days, then they were fixed using paraformaldehyde and stained with crystal violet. The percentage of the area covered by stained cell colonies was evaluated using the ImageJ software.

### RNA isolation and qRT-PCR

Total RNA was extracted from cells or human tissues by using Trizol reagent.1ug RNA was used to synthesize cDNA using PrimeScript^TM^ RT reagent kit according to manufacturer's instructions and qRT-PCR assays were performed using SYBR premix EX Taq. Primers used are shown in Supplementary Table S1. The result, relative expression of miR-202-5p, was normalized against an internal control (U6) and the fold change of miR-202-5p in cancer tissues were compared to the corresponding adjacent normal tissues.

### RNA interference and virus infection

PLKO.1 based shRNA or control vector were co-transfected into HEK293T cells with pREV, pGag and pVSVG at the ratio of 2:2:2:1. The lentiviral supernatant was collected 48 hours after transfection, filtered using a 0.45mm filter, and used for the infection of target cells. Stably transduced cells were selected with 1 ug/ml puromycin. The oligonucleotide sequences of shRNAs are shown in Supplementary Table S1.

### Cell cycle analysis

Cell cycle analysis was performed as previously described 31. Briefly, about 1×10^6^ HCT116 cells were harvested after transfection for 48 hours with miR-202-5p mimics or inhibitors. The cells were then washed with cold PBS and fixed overnight in 75% cold ethanol at 4 °C. Fixed cells were treated with 10 ug/mL RNase A and 20 μg/mL propidium iodide in the dark for 30 minutes before the cell cycle distribution was quantified by flow cytometer. The obtained data was analyzed by using the Multicycle software.

### Cell viability analysis

HCT116 cells were transfected with miR-202-5p mimics or inhibitors for the indicated time period. The cells were then harvested using trypsin and centrifuged at 500 rpm for 3 minutes. Then the cells were re-suspended in fresh medium followed by gentle pipetting to break up clumps. The cell viability was measured via the cell counting assay using a Cellometer Cell Counter (Nexcelom Bioscience, U.S.A.).

### Statistical analysis

Statistical analysis was performed using Microsoft Excel software and GraphPad Prism. Two-tailed student's t-test was used to assess differences between experimental groups. A P-value less than 0.05 was considered to be statistically significant (*P < 0.05; **P < 0.01; ***P < 0.001).

## Results

### Aberrant expression of miR-202-5p in colorectal cancer is critical for cell viability

**Figure 1:**
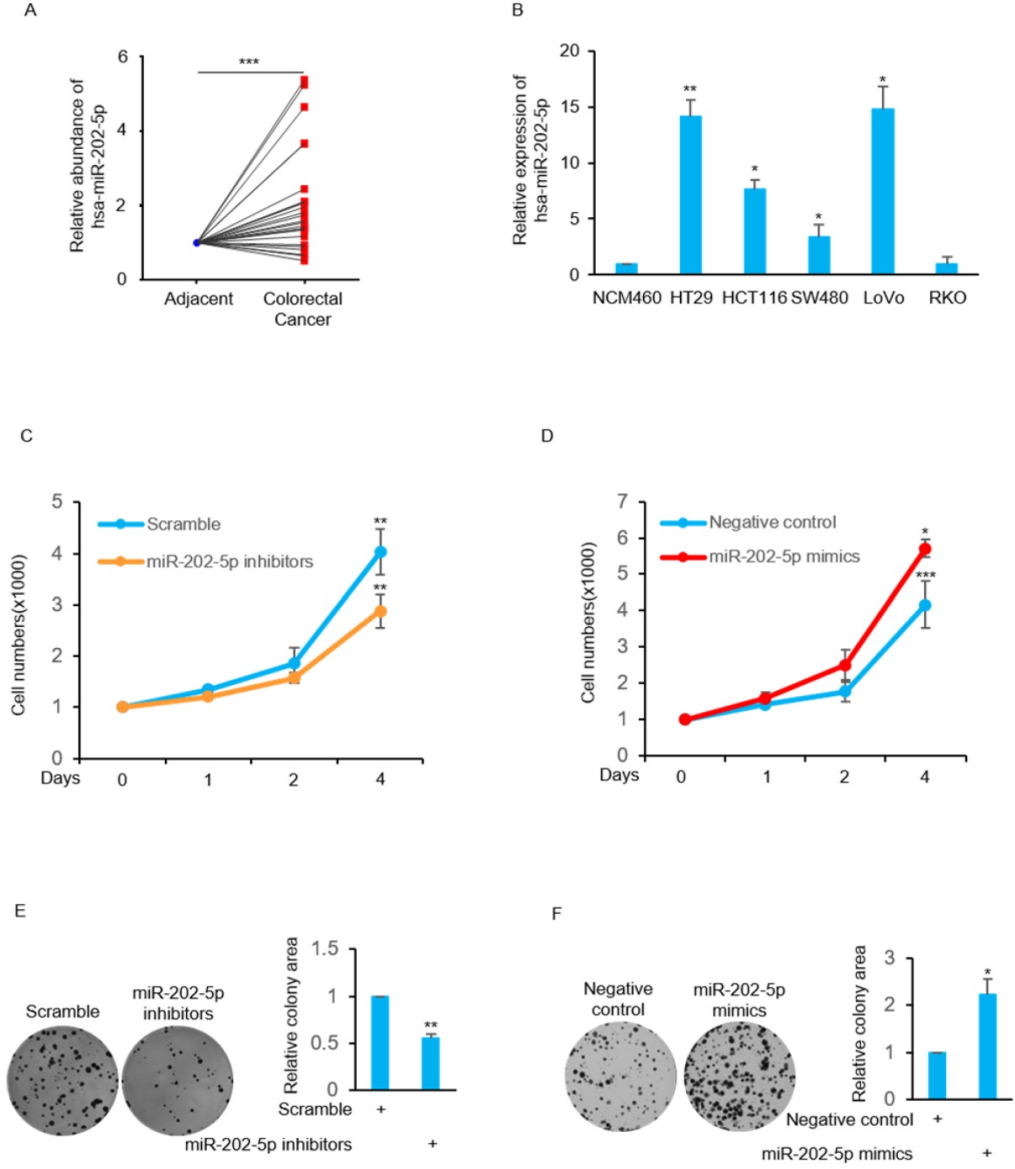
** Aberrant expression of miR-202-5p in colorectal cancer is critical for cell viability. A.** Relative expression of miR-202-5p in 25 pairs of colorectal cancer and adjacent tissues were determined using qRT-PCR. **B.** Relative expression level of miR-202-5p in 5 colorectal cancer cell lines were evaluated according to normal NCM460 cell using qRT-PCR. **C-D.** HCT116 cells were transfected with miR-202-5p inhibitors (C) or mimics (D), then cell numbers were compared with the control group for indicated days. **E-F.** HCT116 cells with inhibition (E) or ectopic expression (F) of miR-202-5p were used to perform colony-formation assays and then the “ColonyArea” were analyzed by Image-J software. All data are representative of three independent experiments.

We examined the relative expression of miR-202-5p by real-time PCR from 25 pairs of human colorectal cancers and corresponding adjacent normal tissues. The results indicated that miR-202-5p expression was significantly elevated in 19 colorectal cancers (Fig. 1A). In addition, the levels of miR-202-5p were exhibited particularly high in colorectal cancer cell lines such as HT29, HCT116, and LoVo when compared with normal cell NCM460 (Fig. 1B).

Next, we focused on examination of the biological function of this high level of miR-202-5p in human colorectal cancers. The obtained results indicate that the cell viability was decreased in HCT116 cells when transfection with miR-202-5p inhibitors was compared with the control group (Fig. 1C). In contrast, introduction of miR-202-5p mimics into HCT116 cells resulted in increased cell viability (Fig. 1D). In addition, the effect of miR-202-5p on cell viability was evaluated in the long-term survival of HCT116 cells in colony-formation assays (Figs. 1E and F). Therefore, these results indicate that miR-202-5p was aberrantly expressed in human colorectal cancers and it is critical for colorectal cancer cell viability.

### MiR-202-5p enhances cell proliferation and cell cycle progression

**Figure 2:**
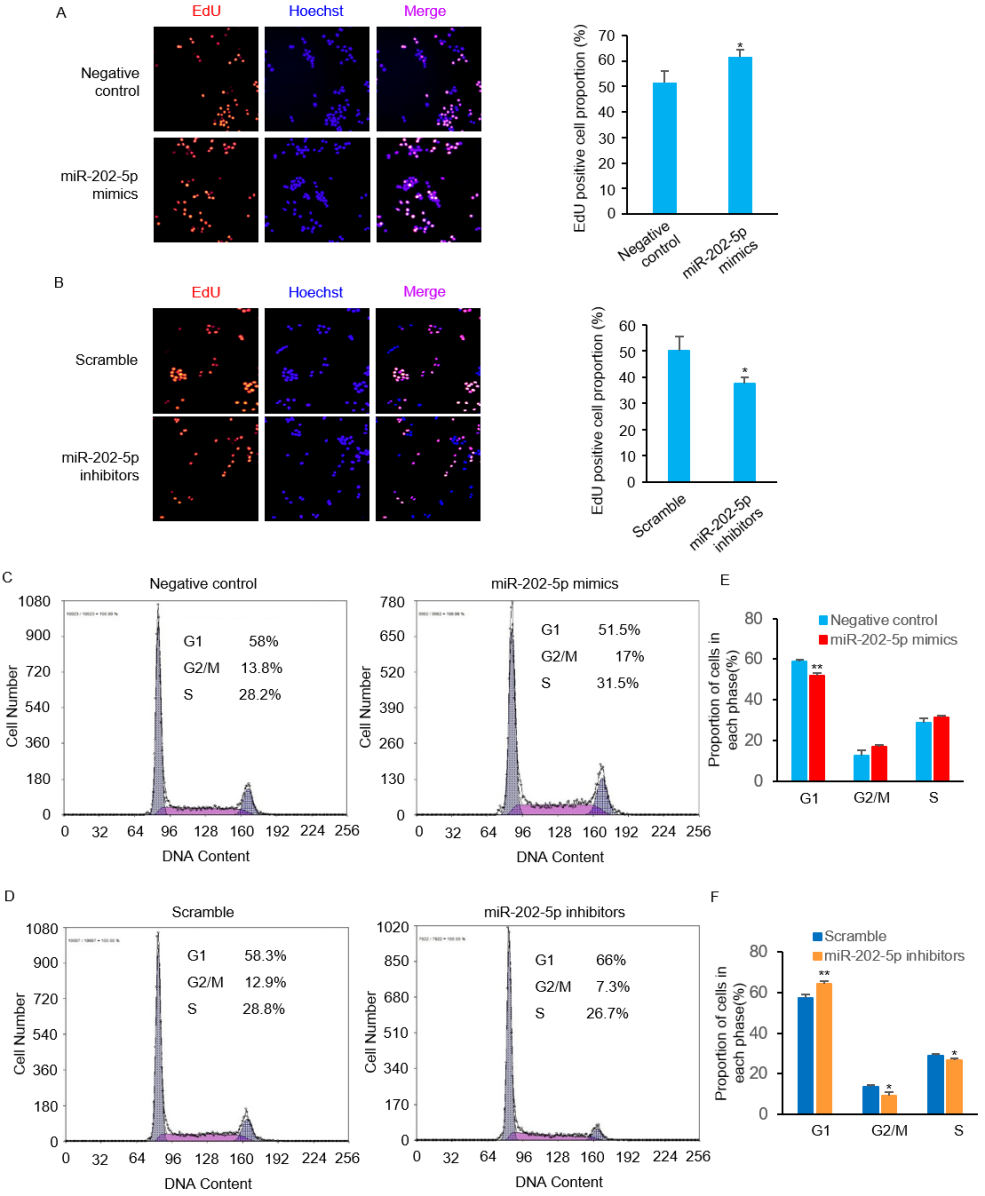
** MiR-202-5p enhances cell proliferation and cell cycle progression. A-B.** HCT116 cells were transfected with miR-202-5p mimics (A) or inhibitors (B) for 48 hours, then were stained with EdU (red), the nuclei were stained with DAPI (blue). The proportion of EdU-positive cells to total DAPI-positive cells was presented and compared with the control group. **C-D.** HCT116 cells were transfected with miR-202-5p mimics (C) or inhibitors (D) as indicated. 48 hours after transfection, cells were stained with propidium iodide and phases of cell cycle were analyzed using flow cytometry. **E-F.** The proportion of cells in G1, G2/M or S phase were analyzed by using Multicycle software. All data are representative of three independent experiments.

We then performed EdU incorporation assays to further confirm the pro-survival role of miR-202-5p. Consistent with the viability changes observed in prior experiments (Figs. 1D and F), introduction of miR-202-5p mimics enhanced the proliferation of HCT116 cells (Fig. 2A). On the other hand, the inhibition of miR-202-5p decreased cell proliferation (Fig. 2B). In addition, flow cytometric analysis showed that introduction of miR-202-5p mimics decreased the percentage of HCT116 cells in the G1 phase, while the percentage of cells in the S and G2 phases was increased (Figs. 2C and E). Consistently, the contrary effect was observed with introduction of anti-miR-202-5p (Figs. 2D and F). These results suggested the acceleratory effect of miR-202-5p on cell cycle progression.

### MiR-202-5p directly targets PTEN and increases Akt activation

**Figure 3:**
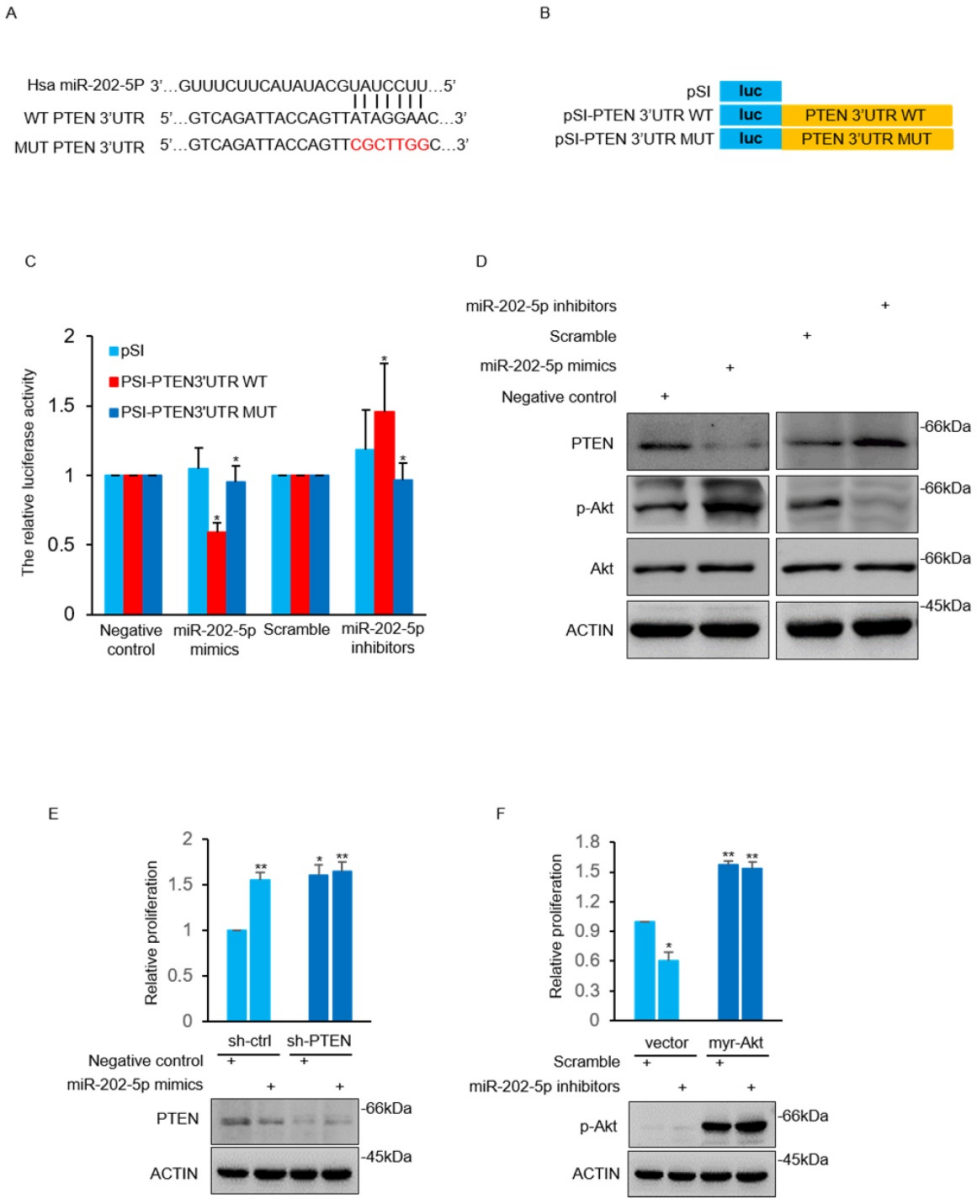
** MiR-202-5p directly targets PTEN and increases Akt activation. A.** Illustration of base pairing of miR-202-5p and wild-type or mutant 3ʹ UTR of PTEN. Substitution of the bases in the mutant 3ʹ UTR of PTEN are shown in the red font. **B.** Schematic illustration of pSICHECK2-based luciferase reporter constructs contains the WT or MUT 3ʹ UTR of PTEN as indicated in (A). **C.** HCT116 cells were co-transfected with mimics or inhibitors of miR-202-5p and the luciferase reporter plasmids indicated in (B). The relative reporter activity was detected using luciferase assays 24 hours after transfection. **D.** HCT116 cells were transfected with miR-202-5p mimics or inhibitors for 48 hours, then whole cell lysates were subjected to Western blot analysis with indicated antibodies. **E.** HCT116 cells stably expressing shRNA-ctrl or shRNA-PTEN were transfected with negative control or miR-202-5p mimics for 72 hours, cell viability was then measured by cell number count and the effect of PTEN-knockdown was confirmed using Western blot assay. **F.** HCT116 cells expressing pcDNA or pcDNA-myr-Akt were transfected with scramble or miR-202-5p inhibitors. 72 hours after transfection, cell viability was measured by cell number count and the phosphorylation of Akt was assessed by Western blot analysis. All data are representative of three independent experiments.

The TargetScan 7.2 database was used to search the potential target gene of miR-202-5p. A potential miR-202-5p binding fragment was identified in the 3' UTR of PTEN gene with nucleotides 110-116 of the 3' UTR matching to the miR-202-5p “seed” region (Fig. 3A). Luciferase reporter assays were conducted with plasmids that contain the wildtype 3'UTR of PTEN as well as the indicated mutant (Fig. 3B). We found that induction of miR-202-5p mimics significantly suppressed the relative activity of the wild-type reporter but not the mutated one. In addition, introduction of miR-202-5p inhibitors increased the wild-type reporter activity which indicated that 3' UTR of PTEN was inhibited by endogenous miR-202-5p (Fig. 3C). Furthermore, miR-202-5p mimics or inhibitors were introduced into HCT116 cells to determine whether miR-202-5p indeed regulates PTEN expression. The results indicated that introduction of miR-202-5p mimics down-regulated the PTEN protein level, whereas the miR-202-5p inhibitors showed the opposite effect when compared with the control (Fig.3D). These results suggested that PTEN is a direct target of miR-202-5p.

Previous studies have reported that PTEN induces Akt termination by dephosphorylating phosphatidylinositol (3, 4, 5) trisphosphate (PI(3,4,5)P3) 33, 34. As anticipated, miR-202-5p mimics increased Akt activation in HCT116 cells in contrast to decreased Akt activation by miR-202-5p antagomirs (Fig. 3D). We next sought to determine whether PTEN mediated Akt activation was involved in miR-202-5p induced cell proliferation. The obtained results showed that the effect of miR-202-5p mimics on cell proliferation was significantly attenuated in HCT116 cells after stable knockdown of PTEN (Fig. 3E). Similarly, introduction of myr-Akt, an active form of Akt, into HCT116 cells prevented the growth-inhibition effect of miR-202-5p antagomirs (Fig. 3F). Thus, PTEN functions as a downstream target of miR-202-5p in regulation of Akt activation, which is responsible for miR-202-5p mediated cell proliferation.

### MiR-202-5p is transcriptionally regulated by c-Myc

**Figure 4:**
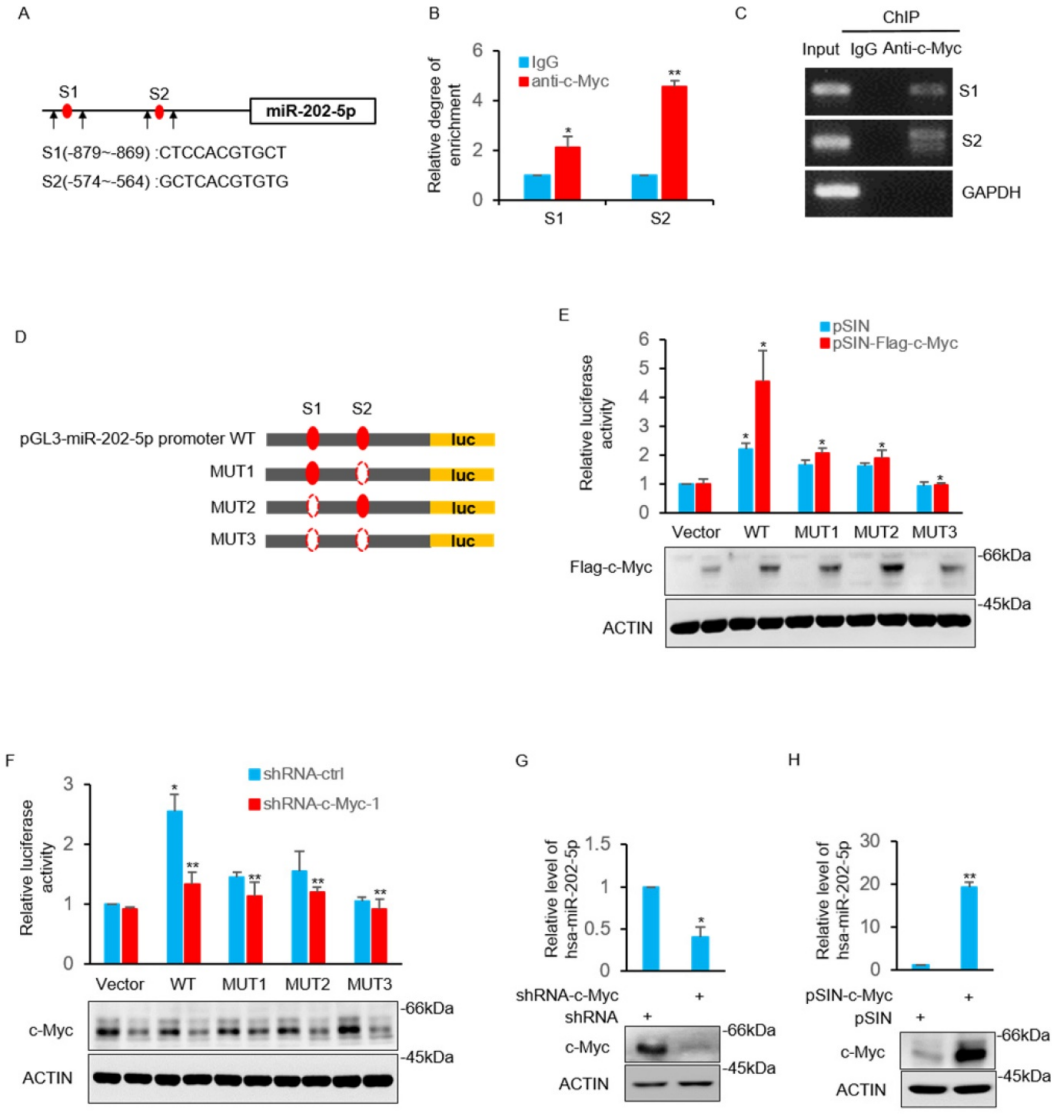
** MiR-202-5p is transcriptionally regulated by c-Myc. A.** Schematic diagram of miR-202-5p promoter and there are two putative c-Myc binding region located in the upstream of the translational start site. Black arrows indicate primers used for PCR in (B) and (C). **B-C.** ChIP assays were conducted in HCT116 cells using anti-c-Myc antibodies or IgG control. ChIP products for miR-202-5p promoter along with GAPDH as a negative control was amplified by qPCR (B) or semi-quantitative RT-PCR (C). **D.** Schematic illustration of pGL3-basic based reporter constructs used for examining the transcriptional activity of miR-202-5p promoter response to c-Myc. Dotted lines indicated the deleted binding region. **E.** HCT116 cells expressing pSIN or pSIN-flag-c-Myc were co-transfected with the indicated reporter constructs and Renilla luciferase plasmids. 24 hours later, relative luciferase activity was detected by luciferase analysis and the successful expression of flag-c-Myc was examined by Western blot analysis. **F.** Luciferase reporter assays were conducted as per (E) to evaluate the effects of c-Myc knockdown. **G.** HCT116 cells were transduced with the shRNA-ctrl or shRNA-c-Myc and miR-202-5p expression was examined using qPCR. **H.** HCT116 cells were transduced with pSIN or pSIN-c-Myc and miR-202-5p expression was evaluated using qPCR. All data are representative of three independent experiments.

The study then sought to determine the underlying mechanism of how miR-202-5p is up-regulated in colorectal cancers. Bioinformatics analysis of the miR-202-5p promoter sequences revealed that two putative c-Myc binding regions (nucleotides -879~-869 and -574~-564) located in the upstream of miR-202-5p translational start site (Fig. 4A). The direct binding of the endogenous c-Myc to these two promoter regions was confirmed by the subsequent chromatin immunoprecipitation (ChIP) assays (Figs. 4B and C). Luciferase reporter plasmids containing the wildtype promoter (nucleotides -1070~-381) of miR-202-5p and three mutant promoters, in which binding regions 1 and 2 were either singly (mut1 and mut2) or doubly (mut3) deleted, were constructed to further ascertain whether these potential c-Myc binding sites were responsive to c-Myc (Fig. 4D). Transfection of c-Myc enhanced the transcriptional activity of the wild-type promoter whereas deletion both of the two binding regions completely abolished the increase in reporter activity. In addition, mutant promoters (mut1 and mut2) with one of the binding regions deleted exhibited a relatively weak transcriptional activity (Fig. 4E). In contrast to overexpression, stable knockdown of c-Myc resulted in decreased the transcriptional activity of the miR-202-5p promoter (Fig. 4F). Additionally, evaluation of the expression of miR-202-5p in HCT116 cells indicated that the knockdown of c-Myc resulted in a significant decrease in miR-202-5p expression, while the enforced expression produced the opposite phenotype (Figs. 4G and H, respectively). Taken together, these results suggested that miR-202-5p is transcriptionally regulated by c-Myc.

### Clinical relevance of the c-Myc-miR-202-5p-PTEN-pAkt axis in colorectal cancer

**Figure 5:**
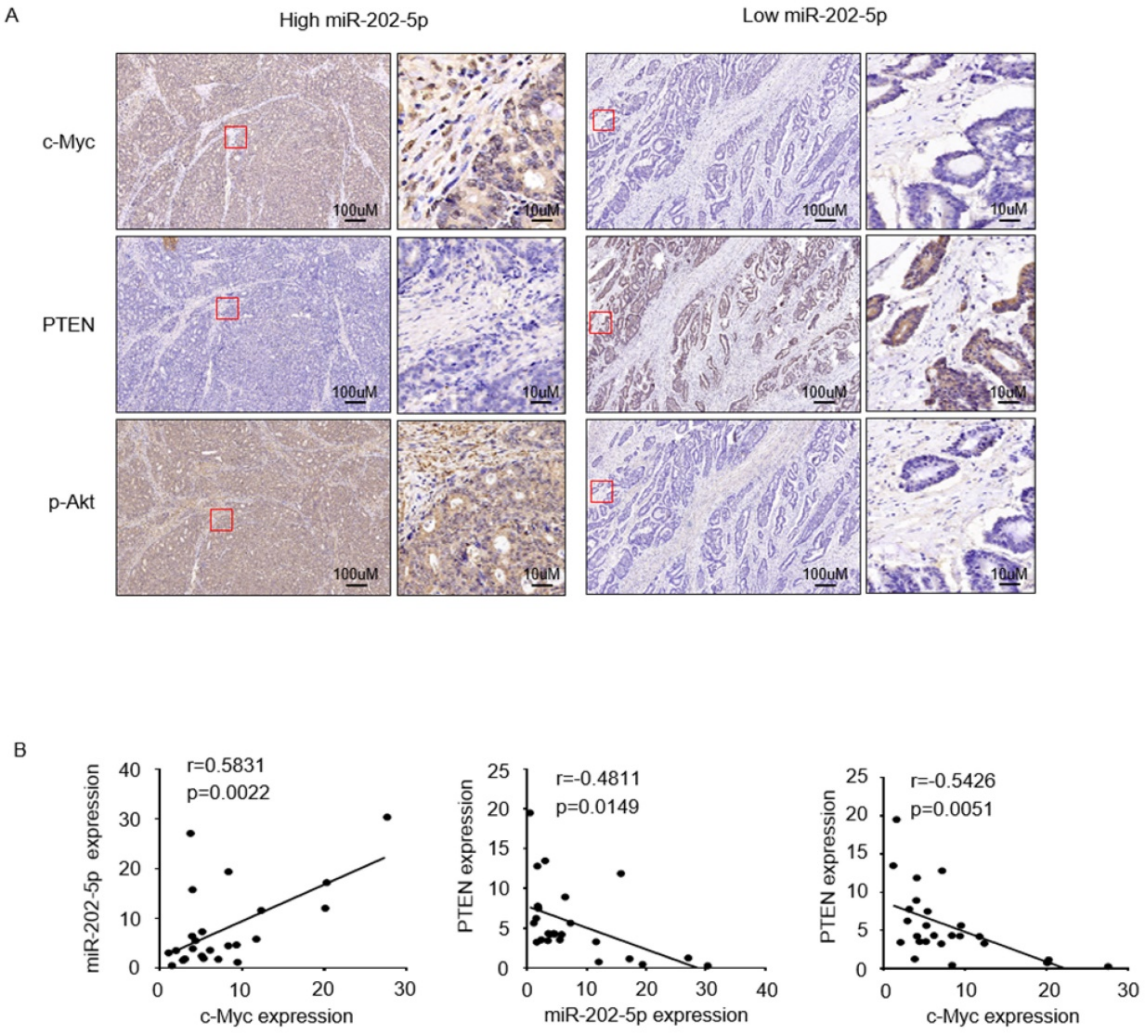
** Clinical relevance of the c-Myc-miR-202-5p-PTEN axis in colorectal cancer. A.** Colorectal cancer tissues from Figure 1A with relatively low (<1-fold change, n=6) and high (>1-fold change, n=19) levels of miR-202-5p expression were subjected to immunohistochemistry analysis with anti-c-Myc, anti-PTEN, and anti-p-Akt antibodies. Representative photomicrographs of IHC were presented. **B.** Correlation analyses conducted among the expression of miR-202-5p, c-Myc, and PTEN respectively, in 25 colorectal cancer tissues. R, Pearson correlation coefficients (r) and p values are shown.

To further examine if the expression of miR-202-5p was correlated with the c-Myc-PTEN-pAkt axis in human colorectal cancers, we divided 25 tumor samples into two groups, relatively low (<1 fold change, n=6) and high (>1 fold change, n=19) levels of miR-202-5p, according to the obtained qRT-PCR results in Figure 1A. Immunohistochemistry analysis indicated that colorectal cancers with high miR-202-5p expression displayed significantly high levels of c-Myc, low levels of PTEN, and high levels of pAkt, while low miR-202-5p expression was associated with less c-Myc and pAkt and more PTEN (Fig. 5A). Correlation analyses also established that miR-202-5p expression was positively correlated with expression of c-Myc, and PTEN expression was negatively correlated with miR-202-5p and c-Myc expression (Fig. 5B).

## Discussion

Deregulation of miR-202-5p has been shown in multiple-types of tumors 14, 17, but its molecular mechanism is still unclear. This study has provided evidences which indicate that the increase of miR-202-5p in CRC appeared to be due to the transcriptional up-regulation mediated by the transcription factor c-Myc. To the best of our knowledge, this is the first study which has reported that c-Myc could directly bind to the “binding site” in the promoter region of miR-202-5p thereby enhancing it expression. Therefore, this study uncovered a novel mechanism for miR-202-5p overexpression in CRC.

Several studies have reported that miR-202-5p is up-regulated in multiple types of cancers, including neuroblastoma, testicular tumors, and breast cancers 12, 17, 18. This study found that miR-202-5p was aberrantly overexpressed and inhibition of miR-202-5p significantly decreased cell proliferation in CRC, which is consistent with previous studies. All these evidences support an oncogenic role of miR-202-5p. Despite our clear evidence showing a pro-tumorgenesis role of miR-202-5p in colorectal cells, miR-202-5p has been reported to inhibit proliferation and invasion by targeting UHRF1 and SMARCC1 in the same cells 35, 36.

In addition, studies have also reported that miR-202-5p is down-regulated in several types of cancers, and functions as a tumor-suppressive regulator in osteosarcoma, bladder cancer, and chronic myeloid leukemia 8,15,16. Furthermore, the overexpression of miR-202-5p induced apoptosis in goat granulosa cells 13. This discrepancy can be attributed to the fact that functional consequences of miRNA expression are tissue and cell type specific 37, 38. Moreover, this may be a controversial issue that needs further study. Therefore, further studies are required to determine whether any other factors are involved in specific regulation of miR-202-5p in colorectal cancer cells.

Aberrant activation of pro-survival signaling pathways is critical for cancer development 20. Among these dysregulated signaling, the PI3K/Akt pathway has been shown to play a particularly important role in CRC 19. Many genetic and epigenetic alterations in CRC, such as amplification of EGFR, mutation of KRAS and loss of PTEN, contributed to the activation of the PI3K/Akt pathway 39. PTEN has been well recognized as a potent tumor suppressor, which was mainly due to its inhibitory effect against the PI3K-Akt pathway 40. Interestingly, in this study we identified PTEN as a novel target of miR-202-5p. Notably, previous studies have reported that several miRNAs participated in the regulation of cancer by targeting PTEN 25, 26. Taken together, these findings indicated that miRNAs mediated post-transcriptional regulation is critical for PTEN expression.

Another interesting discovery in this study was that miR-202-5p promotes tumorigenesis by mediating the “cross-talk” between the oncogenic-protein c-Myc and the tumor-suppressive regulator PTEN. We demonstrated that miR-202-5p expression was elevated in the majority of paired CRC samples (19 of 25). In addition, the elevated expression of miR-202-5p correlated with a corresponding increase in the expression of c-Myc and decreases in the expression of PTEN. This result was consistent with the findings of a prior study which reported that PI3K/Akt signaling was activated in approximately 40% of colorectal cancers 19. Therefore, our findings suggested that c-Myc-miR-202-5p-PTEN axis plays an important role in promoting tumorigenesis and exhibited miR-202-5p is a valuable target in CRC therapy.

## Supplementary Material

Supplementary table S1.
