# Supplementary material for: MiRNA-202-5p promotes Colorectal Carcinogenesis through suppression of PTEN
Source: J Cancer. 2021 Mar 31;12(11):3154–63. doi: 10.7150/jca.56186 (PMC8100819; doi:10.7150/jca.56186)
Supplement: Supplementary file 1 — Supplementary table S1. [file jcav12p3154s1.pdf]

Supplementary table 1

| Oligonucleotide sequences of shRNAs | | |
|---|---|---|
| **shRNAs** | **Sequence (5'-3')** | |
| shRNA-c-Myc1 | CAGTTGAAACACAAACTTGAA | |
| shRNA-c-Myc2 | CCTGAGACAGATCAGCAACAA | |
| shRNA-PTEN1 | CCACAGCTAGAACTTATCAAA | |
| shRNA-PTEN2 | CTAGAACTTATCAAACCCTTT | |
| **Primers used in qRT-PCR & Semi-quantitative RT-PCR analysis** | | |
| **Name** | **Sequence (5'-3')** | |
| | **Forward** | **Reverse** |
| QRT-β-actin | GTGGCCGAGGACTTTGAT | CCTGTAACAACGCATCTCAT |
| RT-miR-202-5p | CTCAACTGGTGTCGTGGAGTCGGCAATTCAGTTGAGCAAAGAAG | |
| RT-U6 | GCTTCACGAATTTGCGTGTCAT | |
| QRT-U6 | CTCGCTTCGGCAGCACA | AACGCTTCACGAATTTGCGT |
| QRT-miR-202-5p | ACACTCCAGCTGGGTTCCTATGCATATACT | TGGTGTCGTGGAGTCG |
| ChIP-GAPDH | TACTAGCGGTTTTACGGGCG | TCGAACAGGAGGAGCAGAGAGCGA |
| ChIP-S1 | TCCGACCCTGCACTCTTG | GCATCAGGCGGACACTGG |
| ChIP-S2 | GCTGAGCTGGGCAGGCCC | CCTTATCTCCCCACGCGC |
| **Antibodies** | | |
| β-actin (WB 1:1000) | CST | Cat#3700 |
| Flag-Tag (WB 1:2500) | Sigma | Cat#F3040 |
| PTEN (WB 1:1000, IHC:1:200) | proteintech | Cat#60300-1-Ig |
| p-Akt (WB 1:1000, IHC:1:400) | proteintech | Cat#66444-1-Ig |
| Akt (WB 1:1000, IHC:1:500) | Cell signaling | Cat#2920S |
| c-Myc (WB 1:1000, IHC:1:500) | Cell signaling | Cat#9402S |
| **Commercial Assays** | | |
| DMEM | Gibco | Cat#12800082 |
| TRIzol | Invitrogen | Cat#AM9738 |
| PrimeScript$^{TM}$ RT reagent Kit | Takara | Cat#RR037A |
| SYBR Green 2xTaq mix | Takara | Cat#RR820A |
| Dual-Luciferase® Reporter Assay System | Promega | Cat#E1910 |
| EdU Assay Kit | Guangzhou RIBOBIO | Cat#C10310-1 |
| cell cycle assay kit | Bestbio | Cat#BB-4104 |
| EZ ChIP™ Chromatin Immunoprecipitation Kit | Millipore | Cat#17-371RF |
